# 37161 Triazole-based reversible inhibitors of spermine oxidase and implications for amelioration of neuronal injury

**DOI:** 10.1017/cts.2021.433

**Published:** 2021-03-30

**Authors:** Amelia Furbish, Patrick Woster

**Affiliations:** Medical University of South Carolina

## Abstract

ABSTRACT IMPACT: This project aims to investigate the impact of spermine oxidase inhibition on amelioration of neuronal injury. OBJECTIVES/GOALS: Our group has recently described a series of triazole-based reversible inhibitors of spermine oxidase (SMOX) (Holshouser et al. 2019). The purpose of the current project is to optimize our most promising inhibitors by structural modification, and to determine whether they can reduce oxidative damage in models of neuronal injury. METHODS/STUDY POPULATION: A small number of SMOX inhibitors have been described in the literature, however, currently available inhibitors lack selectivity for the enzyme and are associated with dose-limiting toxicity. For this project we used multiple medicinal chemistry techniques to synthesize novel triazole-based analogs of our most potent inhibitors as potential SMOX inhibitors. In addition, we plan to utilize virtual and physical screening methods to identify new potential scaffolds. Compounds with demonstrated activity against SMOX via enzymatic assay will then be evaluated in a cell-based model of neuronal injury. In a preliminary study, we investigated the ability of hydrogen peroxide to induce SMOX expression in an SH-SY5Y neuroblastoma cell line using western blot. RESULTS/ANTICIPATED RESULTS: We found that cellular SMOX protein increases in response to hydrogen peroxide exposure in a dose-dependent manner, indicating that this may be a viable cellular model for testing the efficacy of our experimental compounds. To extend these studies, we have developed a SMOX enzymatic assay that will be used for high-throughput screening of commercial libraries, as well as the South Carolina Compound Collection (SC3), which contains 100,000 proprietary, fully annotated analogs. As hits are identified, they will be synthesized and evaluated for potency and selectivity as SMOX inhibitors. The most potent and selective compounds will then be evaluated in our cellular model of neuronal injury. DISCUSSION/SIGNIFICANCE OF FINDINGS: Studies have linked the overexpression of SMOX and the production of associated toxic byproducts with increased susceptibility to excitotoxic stress and neuronal injury. Our objective is to develop potent and selective inhibitors for this enzyme that can serve as chemical probes for elucidating the role of this enzymatic pathway in neuronal injury.

